# Treatment Outcomes in Patients with Metastatic Neuroendocrine Tumors: a Retrospective Analysis of a Community Oncology Database

**DOI:** 10.1007/s12029-018-0160-x

**Published:** 2018-08-18

**Authors:** Maxine D. Fisher, Sonia Pulgar, Matthew H. Kulke, Beloo Mirakhur, Paul J. Miller, Mark S. Walker, Lee S. Schwartzberg

**Affiliations:** 1 Vector Oncology, 6555 Quince Road, Suite 400, Memphis, TN 38119 USA; 2 Ipsen Biopharmaceuticals, Inc., 106 Allen Road, Basking Ridge, NJ 07920 USA; 3 Boston Medical Center, Boston University School of Medicine, One Boston Medical Center Place, Boston, MA 02118 USA; 4 The West Clinic, 100 N Humphreys Boulevard, Memphis, TN 38120 USA

**Keywords:** Somatostatin analog, Neuroendocrine tumor, Community oncology, Overall survival, Progression-free survival

## Abstract

**Purpose:**

Metastatic neuroendocrine tumors (mNETs) are rare, heterogeneous tumors that present diagnostic and treatment challenges, with limited data on the management of mNETs in clinical practice. The present study was designed to identify current diagnostic and treatment patterns in mNET patients treated in the US community oncology setting.

**Methods:**

Patient-level data was collected from medical records of adults with mNETs from the Vector Oncology Data Warehouse, a comprehensive US community oncology network database.

**Results:**

Of the 263 patients included (median follow-up, 22 months; range, 0.1–193.9), 30.4% (80/263) had intestinal tumors, 11.0% (29/263) had pancreatic, and 58.6% (154/263) had tumors of other or unknown location. Progression-free survival (PFS) from the start of first-line therapy differed significantly by tumor grade (log rank *P* = 0.0016) and location (*P* = 0.0044), as did overall survival (OS) (grade, *P* < 0.0001; location, *P* = 0.0068). Median PFS and OS for patients with undocumented tumor grade were shorter than for patients with G1/G2 tumors and longer than patients with G3 tumors. Median PFS and OS for patients with other or unknown tumors were shorter than for patients with intestinal tumors.

**Conclusions:**

While potentially confounded by the high number of patients with other or unknown tumor locations, this retrospective study of patients in a US community oncology setting identified the importance of awareness of tumor grade and tumor location at diagnosis, as these were direct correlates of PFS and OS.

## Introduction

Systemic treatment options for neuroendocrine tumors (NETs) have increased in specificity and efficacy, including interferon alpha, antiangiogenic drugs, mTOR inhibitors, multikinase inhibitors, and peptide receptor radiotherapy [1–3]. Recent randomized, controlled clinical trials have led to an increased number of antiproliferative treatments, including somatostatin analogs (SSAs) and targeted treatments for some patients with advanced, well-differentiated metastatic NETs (mNETs) [4–8]. Treatment with somatostatin analogs (SSAs) has generally been used for symptom control, and these treatments have recently been shown to reduce tumor growth [9].

Poorly differentiated mNETs, which often do not express somatostatin receptors (SSTRs) [10, 11], are generally treated with platinum-based chemotherapy irrespective of primary tumor site [12], while treatment for well-differentiated mNETs is increasingly site specific with antiproliferative therapies, including SSAs for gastroenteropancreatic mNETs [4, 5, 7] and targeted treatment options such as everolimus for pancreatic, gastrointestinal, and lung mNETs and sunitinib for pancreatic mNETs [6, 8, 13–15].

Diagnostic imaging has increased in scope and sophistication in tandem with the increased availability of targeted treatments. Because the majority of well-differentiated NETs express a high density of SSTRs, particularly SSTR subtype 2 [10, 11, 16], imaging with SSTR positron emission tomography/computed tomography (PET/CT) (e.g., ^68^Ga-labeled octreotide (DOTATOC) or octreotate (DOTATATE)) has shown usefulness in identifying NETs of previous unknown primary location [13, 17–21] or recurrent NETs [22]. One study found higher rates of detection of primary tumors with ^68^Ga DOTATOC compared with indium^111^ DTPA in patients with NETs of unknown primary tumor origin [20].

A novel SSTR antagonist (^68^Ga-OPS202) is also being tested as an imaging agent for NETs. Results of a phase 1/2 study showed improved diagnostic contrast and accuracy in imaging GEP-NETs with ^68^Ga-OPS202 compared with ^68^Ga-DOTATOC PET/CT [23, 24]. The better detection of SSTR-positive tumors and primary tumor sites can help improve treatment decisions. In addition, assessment of certain biomarkers, such as CgA or urinary 5-HIAA, may be useful for monitoring disease progression/response to treatment in some NET patients [15, 25].

However, the heterogeneity of NETs and their associated, often nonspecific symptomatology can lead to diagnostic delays, making advanced/metastatic disease more common at the time of diagnosis, particularly in intestinal and pancreatic NET tumors [26, 27]. The importance of accurately identifying tumor grade and primary tumor site is integral to optimizing treatment [12].

For many rare diseases, clinical data from large randomized, controlled trials is often lacking. In certain situations, data describing the characteristics and treatment experience of patients in clinical practice can provide useful, real-world information regarding diagnostic criteria and treatment patterns. For mNETs, these observational data have predominantly come from population-based studies [26, 28–30] and institutional treatment settings [31–34]. In this report, we describe the diagnostic and treatment patterns from a group of patients with mNETs who were treated in a US community oncology setting, to better understand the overall management paradigm for patients with mNETs.

## Patients and Methods

In this retrospective study, we examine information from The Vector Oncology Data Warehouse (VODW), a comprehensive cancer patient database that includes electronic medical record and billing system data for patients who were treated in a US community oncology setting. Patients from the VODW who were ≥ 18 years of age at the time of diagnosis of mNET were eligible for inclusion. Following institutional review board approval of the study protocol, demographic, medical, treatment, and select patient symptom data were collected from the VODW. Clinical Research Nurses (CRNs) verified eligibility and extracted information onto case report forms that were entered into a secured data management system for analysis. Dates of death were recorded from the clinical record or the linked Social Security death index data.

### Assessments

A treatment regimen was defined as one or more anticancer agent given for NET in combination in which: (1) all agents started ≤ 30 days from the start of the first agent, unless the start of an agent > 30 days after the first agent was prespecified as part of the treatment plan; (2) no agent was discontinued and replaced by another ≤ 30 days after the first agent; and (3) no agent was held and then resumed after 42 days. First-line therapy was defined as the first regimen the patient received after diagnosis of mNET. PFS was defined as the time from the start of first-line therapy to documented disease progression or death and overall survival (OS) was defined as the time from initiation of first-line therapy to death. No information regarding safety or tolerability was extracted.

### Statistical Analysis

Statistical comparisons among or between groups were conducted using analysis of variance (ANOVA) for continuous variables and a chi-square or Fisher’s exact test for categorical variables. Kaplan-Meier survival analysis and log-rank test were performed to examine PFS and OS.

## Results

A total of 263 patients with mNETs treated between November 1996 and May 2015 were included in the analysis. Median patient age was 65.0 years (range, 22–92 years), 50.6% of patients were female, and 73.4% were White. Most patients (80.6%) were at stage IV at initial diagnosis. The median duration of follow-up in this study population was 22 months (range, 0.1–193.9 months). The majority of patients in the study (*n* = 118/263, 44.9%) had grade (G)1 (*n* = 95, 36.1%) or G2 (*n* = 23, 8.7%) tumors (Table 1), with 63 patients (24.0%) having G3 tumors. For about one third of all the patients (*n* = 82/263, 31%), tumor grade was not documented. Approximately 50% (132/263) of patients had a documented primary tumor site recorded, and 48% (126/263) had a metastatic tumor site first recorded. Among all 263 patients, 73 (28.3%) had jejunal/ileal/colon (intestinal) tumors, 29 (11.2%) had pancreatic tumors, and more than half (*n* = 154, 58.6%) had other tumors, including bronchopulmonary (*n* = 13), gastric (*n* = 8), rectal (*n* = 7), thymus (*n* = 1), unknown (*n* = 2), or undocumented (*n* = 5). A total of 121 (46.9%) patients were documented as having “other” tumor subtypes, consisting of various primary or metastatic tumor locations, primarily liver (*n* = 60) and mesentery (*n* = 27).

**Table 1:** Clinical characteristics by tumor grade

	Tumor grade			*P* value^a^
	Grade 1/grade 2 (*n* = 118)	Grade 3 (*n* = 63)	Not documented (*n* = 82)	
Stage of disease at initial diagnosis (*n* (%))				
IV	102 (86.4)	51 (81.0)	59 (72.0)	0.0190
III	5 (4.2)	2 (3.2)	2 (2.4)	
II	2 (1.7)	1 (1.6)	0	
I	0 (0.0)	1 (1.6)	0 (0.0)	
Other	0	0	1 (1.2)	
Undocumented	9 (7.6)	8 (12.7)	20 (24.4)	
First documented tumor subtype (*n* (%))^b^				
Primary tumor	63 (53.4)	28 (44.4)	41 (50.0)	0.5070
Metastatic tumor	54 (45.8)	34 (54.0)	38 (46.3)	
Undocumented	1 (0.8)	1 (1.6)	3 (3.7)	
Tumor origin (*n* (%))^b^				
Intestinal^a^	52 (44.1)	8 (12.7)	20 (24.4)	< 0.0001
Pancreatic^a^	7 (5.9)	10 (15.9)	12 (14.6)	0.0573
Other/unknown^a^	61 (51.7)	45 (71.4)	51 (62.2)	0.0309
Primary tumor grade (*n* (%))				
G1: Well differentiated	47 (85.5)	0	–	
G2: Moderately differentiated	8 (14.5)	0	–	
G3: Poorly differentiated	0	26 (100.0)	–	
Metastatic tumor grade (*n* (%))				
G1: Well differentiated	48 (76.2)	0	–	< 0.0001
G2: Moderately differentiated	15 (23.8)	0	–	
G3: Poorly differentiated	0	36 (100.0)	–	
Mention of carcinoid symptoms or carcinoid syndrome^c^ (*n* (%))	75 (63.6)	12 (19.0)	27 (32.9)	< 0.0001
Mention of chromogranin A (CgA) (*n* (%))	81 (68.6)	28 (44.4)	43 (52.4)	0.0036
Mention of 5-hydroxyindoleacetic acid (5-HIAA) (*n* (%))	45 (38.1)	10 (15.9)	31 (37.8)	0.0048

*BMI*, body mass index; *SD*, standard deviation

^a^*P* values derived from an ANOVA or Kruskal-Wallis test for continuous variables and a chi-square or Fisher’s exact test for categorical variables. In instances for which exact computations required a great amount of time and memory, *P* values were estimated by Monte Carlo simulation

^b^The record was checked for documentation of tumor subtype and grade. If documented for a primary tumor, that subtype or grade was recorded. If not, and documentation for a metastatic tumor existed, that subtype or grade was recorded. If neither existed in the record, the subtype or grade was recorded as undocumented. For reporting collapsed tumor subtype, the following groupings were used: intestinal includes jejunal/ileal/colon/duodenal/appendix; other includes bronchopulmonary/gastric/rectal/thymus/stated as unknown/undocumented. Three patients with documented metastatic tumor subtypes had both jejunal/ileal/colon and other as the first documented. For inclusion in models, these patients were categorized as jejunal/ileal/colon (other, *n* = 154)

^c^Categories are not mutually exclusive

### Treatment Analysis

The majority of patients with G1/G2 or undocumented tumor grade received SSA treatment in line 1 (87.4 and 66.2%, respectively) as monotherapy or in combination (Table 2). The majority of patients with G3 tumors received cytotoxic treatment in line 1 of therapy (82.1% [46/63]). There was little utilization of tyrosine kinase inhibitors (*n* = 6, 2.7%) or mTOR inhibitors (*n* = 2, 0.9%) in any patient subgroup population.

**Table 2:** Lines of therapy (collapsed treatment regimens) for overall sample (*N* = 263)

Line	Regimen	Tumor grade			Overall (*N* = 263)
		Grade 1/grade 2 (*n* = 118)	Grade 3 (*n* = 63)	Undocumented grade (*n* = 82)	
1	CT	12 (12.6%)	46 (82.1%)	24 (35.3%)	82 (37.4%)
	SSA	83 (87.4%)	10 (17.9%)	45 (66.2%)	138 (63.0%)
	TKI	3 (3.2%)	2 (3.6%)	1 (1.5%)	6 (2.7%)
	mTOR inhibitor	2 (2.1%)	0 (0%)	0 (0%)	2 (0.9%)
	Overall	95	56	68	219
2	CT	9 (14.5%)	23 (71.9%)	11 (24.4%)	43 (30.9%)
	SSA	53 (85.5%)	10 (31.3%)	33 (73.3%)	96 (69.1%)
	TKI	2 (3.2%)	0 (0%)	1 (2.2%)	3 (2.3%)
	mTOR inhibitor	5 (8.1%)	3 (9.4%)	1 (2.2%)	9 (6.5%)
	Overall	62	32	45	139
3	CT	6 (14.6%)	10 (50.0%)	7 (23.3%)	23 (25.3%)
	SSA	35 (85.4%)	9 (45.0%)	24 (80.0%)	68 (74.7%)
	TKI	1 (2.4%)	1 (5.0%)	0 (0%)	2 (2.2%)
	mTOR inhibitor	5 (12.2%)	1 (5.0%)	0 (0%)	6 (6.6%)
	Overall	41	20	30	91
4	CT	3 (13.6%)	7 (58.3%)	2 (13.3%)	12 (24.5%)
	SSA	20 (90.9%)	5 (41.7%)	14 (93.3%)	39 (79.6%)
	mTOR inhibitor	1 (4.5%)	2 (16.7%)	0 (0%)	3 (6.1%)
	Overall	22	12	15	49
5	CT	1 (8.3%)	3 (50.0%)	3 (25.0%)	7 (23.3%)
	SSA	12 (100.0%)	2 (33.3%)	10 (83.3%)	24 (80.0%)
	TKI	0 (0%)	1 (16.7%)	0 (0%)	1 (3.3%)
	mTOR inhibitor	1 (8.3%)	0 (0%)	1 (8.3%)	2 (6.7%)
	Overall	12	6	12	30

Categories are not mutually exclusive

*CT*, cytotoxics; *mTOR*, mammalian target of rapamycin; *SSA*, somatostatin analog; *TKI*, tyrosine kinase inhibitor

### Progression-Free Survival

**Fig. 1:**
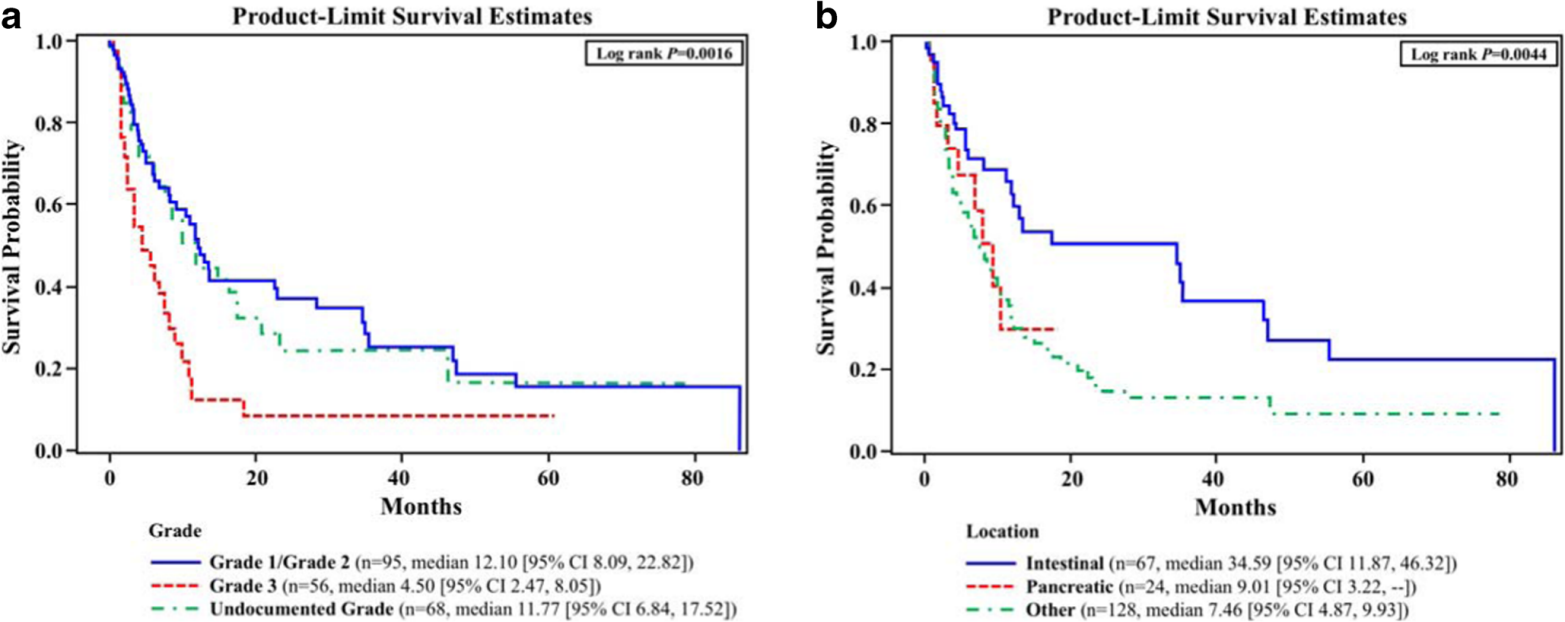
Kaplan-Meier analysis of PFS from start of first-line therapy (*n* = 219). **a** PFS by tumor grade. In the evaluation of PFS by tumor grade, median PFS was 12.1 months for those with G1 or G2 tumors (*P* = 0.225) and 4.5 months for those with G3 tumor. For patients with undocumented tumor grade, there was a median PFS of 11.8 months. **b** PFS by tumor location. In the evaluation of PFS by tumor location, median PFS was 34.6 months for those patients with intestinal tumors (*n* = 67) and 9.0 months for those with pancreatic tumors (*n* = 24). For patients with other tumor location (*n* = 128), there was a median PFS of 7.5 months. Patients with intestinal tumors (*n* = 42) had a median PFS of 34.6 months, with patients with pancreatic tumors (*n* = 6) demonstrating a PFS of 10.4 months. Patients with other or unknown tumor locations (*n* = 47) that were G1 or G2 had a median PFS of 8.1 months

PFS from the start of first-line therapy differed significantly by tumor grade (log rank *P* = 0.0016) and by tumor location (log rank *P* = 0.0044) (Fig. 1a, b). Median PFS from the start of first-line therapy for patients with undocumented tumor grade was shorter than the median PFS for patients with G1/G2 tumors and longer than the median PFS for patients with G3 tumors (Fig. 1a). However, median PFS for patients with other/unknown tumors was shorter than for patients with intestinal or pancreatic tumors**.**

### Overall Survival

**Fig. 2:**
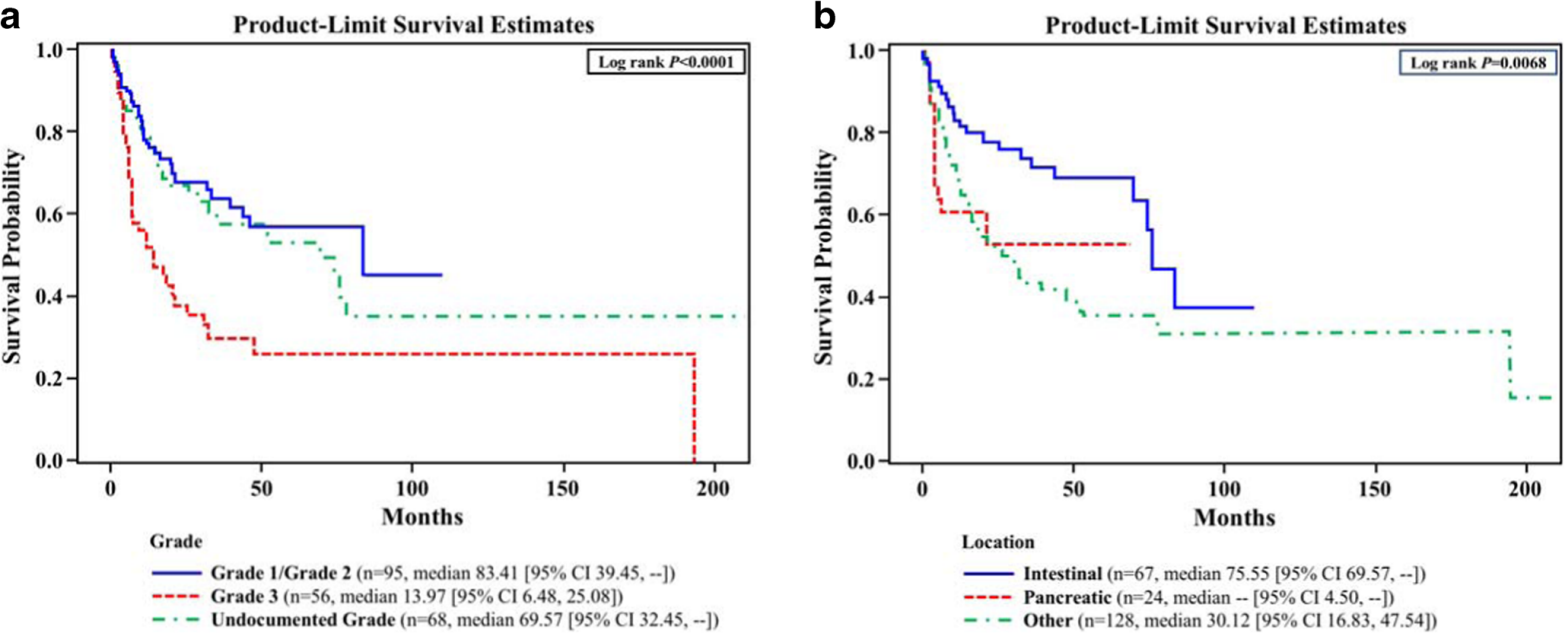
Kaplan-Meier analysis of OS from start of first-line therapy (*n* = 219). **a** OS by tumor grade. In the evaluation of OS by tumor grade, median OS was 83.9 months for those with G1 or G2 tumors, and 12.3 months for those with G3 tumors. Patients with other or unknown tumor grade had a median OS of 71.5 months. In the analysis of OS in patients with G1/G2 tumors, the overall log rank test was statistically significant (log rank *P* = 0.0427). **b** OS by tumor location. When evaluated by tumor type, median OS was 102.8 months for those with intestinal tumors (*n* = 80) and 69.7 months for those with pancreatic tumors (*n* = 29). Patients with other or unknown tumor location (*n* = 152) had a median OS of 32.8 months. The median OS was 102.8 months for those patients with intestinal tumors (*n* = 52), with no OS observed in patients with pancreatic tumors (*n* = 7). Patients with other or unknown tumor location (*N* = 59) had a median OS of 56.9 months.  CI, confidence interval; OS, overall survival; SD, standard deviation. **a** Mean was biased downward because there were censoring times greater than the largest event time. **b **Two patients have missing survival time due to unknown date of mNET diagnosis

OS was able to be calculated for *n* = 261 patients from the overall study population, with a median OS of 56.9 months (based on time from diagnosis of metastatic disease) for the total cohort. OS from the start of first-line therapy differed significantly by both tumor grade (log rank *P* < 0.0001) and tumor location (log rank *P* = 0.0068) (Fig. 2a, b). Median OS from the start of first-line therapy for patients with undocumented tumor grade was shorter than the median OS for patients with G1/G2 tumors and longer than the median OS for patients with G3 tumors (Fig. 2a). However, median OS for patients with other/unknown tumors was shorter than patients with intestinal tumors (Fig. 2b).

## Discussion

Although differences in study designs and study populations make comparisons difficult, the treatment trends and outcomes reported in this analysis of data from the Vector Oncology network were generally in line with previously reported observational studies in institutional and community oncology settings [31, 32, 35, 36]. Specifically, low-grade (G1 or G2) tumors were associated with better survival outcomes than patients with high grade tumors, and patients with primary tumors of the pancreas had worse PFS than patients with intestinal tumors. Some noteworthy findings from the Vector analysis were the high incident rates of patients with undocumented tumor grades and other or unknown tumor locations and their respective outcomes.

In this analysis, the median survival for patients with other/unknown tumor grades (*n* = 68, 69.6 months) fell between that of patients with G1/G2 tumors (*n* = 95, 83.4 months) and G3 tumors (*n* = 56, 14.0 months), with the median OS for patients with other/unknown tumor location (*n* = 128, 30.1 months) being worse than patients with intestinal tumors (*n* = 67, 75.6 months). In general, outcomes for patients with undocumented NET grades were not well reported. In this study, it is possible that patients identified as having undocumented tumor grade may have included a mix of G1/G2/G3 tumors and/or tumors of mixed histology. Yao et al. reported a similar trend in survival, where patients with tumors of mixed histology had shorter median survival compared with those who had G1 or G2 tumors [29]. Likewise, in a single-center institutional study, the adjusted median OS for patients with other or unknown primary tumors (other defined as not small bowel or pancreas) was similar to that of patients with advanced pancreatic NETs (defined as metastatic disease), and the median OS in both of these groups was shorter than the median OS for patients with midgut tumors [31]. These results suggest that patients in these studies with unknown primary tumor category were likely represented by a mixture of pancreatic and intestinal tumors. Although there may be additional unknown factors that contributed to the worse survival observed in these patients, indirect support for this theory comes from another observational study conducted in patients with “mid-gut” mNETs that reported no significant difference in median survival between patients with known primary tumor site (110 months) and unknown primary tumor site that the investigators suspected to be of intestinal origin (73 months) [36]. The results of the Cox Model indicated no statistically significant differences in PFS by tumor location when adjusting for other factors.

The rates of undocumented tumor grade (31%) and other/unknown tumor location (58.6%) from this analysis are notable, as both are important factors in the treatment and management of mNETs [12]. Similar rates of undocumented tumor differentiation have been reported in community oncology networks (41.5%) [35]; however, single-center institutional studies have reported lower rates ranging from 3.4 to 11% [31, 34]. Other/unknown primary tumor locations have been reported at variable rates ranging from 11 to 39% [29, 31, 36, 37]. Of note, 48% of patients in the Vector database did not have a documented primary tumor site reported at diagnosis; rather, these patients had a metastatic tumor site recorded at diagnosis. However, for the survival analysis, the “other/unknown” tumor category included both primary and metastatic tumor sites, which may have impacted the outcomes. Nonetheless, these findings raise important questions regarding potential factors that may contribute to undocumented tumor grade and tumor location. Reasons for undocumented tumor grade may include limitations with current pathology assays or classification systems, particularly when mixed histology is encountered. There is the potential that obtaining a biopsy may not have been feasible in some patients. Identification of the primary tumor can also be challenging in some patients with NETs and may reflect a lack of sensitivity in diagnostic imaging, particularly for smaller tumors. Future research should be conducted to clarify whether tumor histology is routinely obtainable across oncology practice settings and to determine what diagnostic imaging and examination methods are being used.

In this analysis, most of the patients with undocumented tumor grade received SSA treatment as first-line therapy, similar to patients with G1 or G2 tumors. In the absence of diagnostic imaging information regarding tumor grade, clinicians may be likely to choose treatment based on patient symptoms as well as their own experience. However, high-grade, poorly differentiated tumors are more aggressive in nature and do not typically respond to SSA or targeted therapies, which makes it essential to distinguish well-differentiated from poorly differentiated NETs at diagnosis [38]. The implications of unknown primary tumor locations are also important in terms of opportunities for surgical resection and potential targeted treatments for well-differentiated NETs depending on tumor site that can improve outcomes [15]. Although further analysis of the subset of patients with other or unknown tumor location was beyond the scope of this study, these findings emphasize the importance of more accurate diagnostic methods to promote earlier diagnosis, not only for NETs.

Biomarkers such as CgA and urinary 5-HIAA may be used for monitoring disease activity in some NET patients [15, 25]. Unfortunately, information about these two biomarkers was not well reported in the current study and could not be analyzed. A study conducted across oncology practices in the USA reported changes in biomarkers post-treatment; however, the incidence of unknown or missing information was frequent, occurring in 65.1 and 67.3% of patients for CgA and urinary 5-HIAA, respectively [35]. Additional reporting on the use of specific biomarkers in patients with NETs treated in community and tertiary care settings may provide further insight into current patient monitoring practices.

Because of the retrospective nature of this analysis and variable follow-up time, there is a potential risk for misclassification or selection bias. In addition, as in all observational studies, this analysis does not allow us to identify any potential causal relationships. Despite these limitations, results from this analysis found that the median survival rates in patients with G1 or G2 tumors (the majority of whom were treated with SSAs as monotherapy or in combination) or in patients with G3 tumors (who received cytotoxic treatment) were generally similar to those in previously reported studies.

A substantial proportion of patients in this analysis had undocumented tumor grade or unknown tumor location, indicating that many patients are being treated despite a lack of information that might improve the care they receive. The large number of patients with tumor location and tumor grade classified as other or unknown may reflect areas of variability in clinical practice in this setting, possibly due to the availability of different resources in different treatment setting. Additional prospective study is warranted to help clarify and further explore these findings.

## Data Availability

The datasets generated during and/or analyzed during the current study are not publicly available due to contractual limitations regarding rights to use of the data but may be available from the corresponding author on reasonable request.
